# ROTS: reproducible RNA-seq biomarker detector—prognostic markers for clear cell renal cell cancer

**DOI:** 10.1093/nar/gkv806

**Published:** 2015-08-11

**Authors:** Fatemeh Seyednasrollah, Krista Rantanen, Panu Jaakkola, Laura L. Elo

**Affiliations:** 1 Turku Centre for Biotechnology, University of Turku and Åbo Akademi University, Turku, FI-20520, Finland; 2 Department of Mathematics and Statistics, University of Turku, Turku, FI-20014, Finland; 3 Department of Medical Biochemistry, University of Turku, Turku, FI-20014, Finland; 4 Department of Oncology and Radiotherapy, Turku University Hospital, FI-20520 Turku, Finland

## Abstract

Recent comprehensive assessments of RNA-seq technology support its utility in quantifying gene expression in various samples. The next step of rigorously quantifying differences between sample groups, however, still lacks well-defined best practices. Although a number of advanced statistical methods have been developed, several studies demonstrate that their performance depends strongly on the data under analysis, which compromises practical utility in real biomedical studies. As a solution, we propose to use a data-adaptive procedure that selects an optimal statistic capable of maximizing reproducibility of detections. After demonstrating its improved sensitivity and specificity in a controlled spike-in study, the utility of the procedure is confirmed in a real biomedical study by identifying prognostic markers for clear cell renal cell carcinoma (ccRCC). In addition to identifying several genes previously associated with ccRCC prognosis, several potential new biomarkers among genes regulating cell growth, metabolism and solute transport were detected.

## INTRODUCTION

The recent comprehensive assessments of the RNA-seq technology provide important guidelines to produce high-quality RNA-seq data sets (1–4). The overall results from these community efforts demonstrate reproducibility of RNA-seq platforms and data analysis strategies for quantifying gene expression levels. Although these evaluations involved controlled experiments that are rather far from actual clinical applications, they strongly support that the RNA-seq technology can produce data that is of sufficient quality to many biomedical applications.

In addition to producing accurate estimates of gene expression levels, the utility of the RNA-seq technology depends on the availability of rigorous tools for downstream analysis of these data such as quantifying differences between sample groups. This part still lacks well-defined best practices. Although a number of advanced statistical methods have been developed (e.g. edgeR (5,6), DESeq (7), baySeq (8), Cuffdiff2 (9)), several studies demonstrate that their performance depends strongly on the data under analysis and there is no ‘one fits all method’ that would always perform well (10–12). This compromises practical utility in real biomedical and clinical studies that aim to identify reliable biomarkers for diagnosis, prognosis or treatment of patients.

To address the challenge of selecting a suitable statistic, we propose to use a data-adaptive procedure, named ROTS (Reproducibility Optimized Test Statistic). It determines an optimal test statistic directly from the data by maximizing the reproducibility of the detections across bootstrap samples (refer to MATERIALS AND METHODS for details). The utility of reproducibility optimization in microarray studies of gene expression has been demonstrated (13,14). In this study the reproducibility optimization is shown to significantly improve the reliability of differential expression detection in RNA-seq data for the first time. An R-package implementing ROTS is available at http://www.btk.fi/research/research-groups/elo/software/rots/.

## MATERIALS AND METHODS

### Data sets

#### Spike-in data set

The spike-in data set was generated by Rapaport et al. (11) and the expression files were downloaded from GEO with the accession number GSE49712. The selected samples were part of SEQC (MAQC-III) project extracting from Stratagene Universal Human Reference RNA (UHRR) and Ambion Human Brain Reference RNA (UBRR). The samples were divided into two distinct experimental groups *A* and *B* with five technical replicates per group. All the replicates were enriched with 92 synthetic polyadenylated oligonucleotides introduced by the External RNA Control Consortium (ERCC) (15) to validate the differential expression findings. The ERCC spike-in controls were spiked to have 0.5-, 0.67-, 1- or 4-fold changes between the mixture groups *A* and *B*. All the samples were sequenced using Illumina HiSeq2000 platform and produced paired-end reads of length 100 bp. The reads were mapped and assembled using TopHat (v.2.0.3) (16) and UCSC hg19 as the genome reference. HTSeq (v.0.5.p3) was applied for gene expression abundance estimation (17).

#### TCGA clear cell renal cell carcinoma (ccRCC) data set

The ccRCC data set was published by The Cancer Genome Atlas (TCGA) (18). All the biospecimens were collected from patients with ccRCC diagnosis and kidney as primary site before any treatment procedures. The mRNA expression files were downloaded from the TCGA website with IlluminaHiSeq_RNASeqV2 platform code (see https://tcga-data.nci.nih.gov/tcga/). The demographics data and clinical features were available in the supplementary tables of the original study (18). In total, 448 patients had mRNA expression data available in TCGA, among which 442 patients had the necessary clinical values available and were used in the analysis.

#### ccRCC validation data set

The ccRCC prognostic findings were validated in an independent RNA-seq data set published by Sato et al. (19). The mRNA libraries had been sequenced into 100 bp paired-end reads using Illumina HiSeq2000 platform. The alignment files were downloaded from European Genome-phenome Archive (EGA) using the accession number EGAS00001000509 (Data Set ID: EGAD00001000597). The expression levels were estimated using HTSeq (v.0.6.1) package and UCSC hg19 genome reference. The clinical features were obtained from the original publication (19). Those 93 samples which included complete clinical data were used in the analysis.

### Preprocessing procedures: TMM normalization and Voom transformation

The necessity of normalization has been proved in RNA-seq studies (20,21). Accordingly, the Trimmed mean of M values (TMM) method implemented in Bioconductor edgeR package was used to normalize the expression levels. The Voom transformation implemented in Bioconductor Limma package was applied to transform the expression levels suitable for further differential expression testing, following the recommendation by Law et al. (22).

### ROTS differential expression testing

Making *a priori* assumptions about data set distributions contradicts the observed biological variation in real RNA-seq experiments. To eliminate biases, we propose to learn an appropriate test statistic directly from the data, building on our data-adaptive reproducibility optimization procedure ROTS (Reproducibility Optimized Test Statistic) (13). The input of ROTS is a count matrix with genomic features as rows and samples as columns. Genomic feature can refer to a gene, a transcript or an exon but it is called gene throughout this manuscript for convenience. The aim of ROTS is to rank the genes according to their differential expression. For each data set, the ranking statistic is determined by maximizing the reproducibility of the gene rankings in bootstrapped data sets.

Let us denote by $x_{gi}^j$ the normalized read count of gene $g$ in sample $i$ from condition$j$. The mean and variance of gene $g$ within each condition is defined as

\begin{equation*} \bar x_{g}^j = \frac{1}{{n_j }}\mathop \sum \nolimits_{i = 1}^{n_j } x_{gi}^j \;{\rm and}\;\left( {s_g^j } \right)^2 = \frac{1}{{n_j - 1}}\mathop \sum \nolimits_{i = 1}^{n_j } \left( {x_{gi}^j - \bar x_{g}^j } \right)^2 ,\end{equation*}

where $n_j$ is the number of samples in condition *j*. The gene rankings are estimated using a family of modified *t*-statistics:

\begin{equation*} d_\alpha \left( g \right) = \frac{{\left| {\bar x_{g}^1 - \bar x_{g}^2 } \right|}}{{\alpha _1 + \alpha _2 s_g }},\quad \alpha _1 \in \left[ {0,\left. \infty \right)} \right.,\quad \alpha _2 \in \left\{ {0,1} \right\}.\end{equation*}

Here, $\alpha _1$ and $\alpha _2$ are common to all genes and

\begin{equation*} s_g = \sqrt {\left( {\frac{1}{{n_1 }} + \frac{1}{{n_2 }}} \right)\frac{{\left( {n_1 - 1} \right)\left( {s_g^1 } \right)^2 + \left( {n_2 - 1} \right)\left( {s_g^2 } \right)^2 }}{{n_1 + n_2 - 2}}} \end{equation*}

is the pooled standard error of gene $g$ across the conditions. Specific choices of the parameters $\alpha$ determine the ROTS statistic. For instance, the special case of $\alpha _1 = 0$ and $\alpha _2 = 1$ corresponds to the ordinary *t*-statistic. The other special cases include signal log-ratio ($\alpha _1 = 1$ and $\alpha _2 = 0$) or SAM-statistic (23) ($\alpha _2 = 1$ and $\alpha _1$ a percentile of the standard deviations). In ROTS the parameters $\alpha$ are not predefined but they are determined by a reproducibility optimization procedure. The aim is to ensure appropriate accuracy of variance estimation, which is considered as the main challenge in RNA-seq data analysis.

The optimization of $d_\alpha \left( g \right)$ is based on maximizing its reproducibility through bootstraps. Pairs of bootstrapped data sets $D_1^b$ and $D_2^b$ are sampled from the source data set $D$ preserving the same sample size and sample labels (sampling with replacement within the groups). The reproducibility is then computed as the average overlap of the *k* most top ranked genes ordered by applying the test statistic $d_\alpha$ across *B* pairs of bootstrap data:

\begin{equation*} R_k {\rm }\left( {d_\alpha } \right) = \frac{1}{B}\mathop \sum \limits_{b = 1}^B R_k^b \left( {d_\alpha } \right).\end{equation*}

For the optimization, a *z*-type statistic is used defined as

\begin{equation*} Z_k \left( {d_\alpha } \right) = \frac{{R_k \left( {d_\alpha } \right) - R_k^0 \left( {d_\alpha } \right)}}{{s_k \left( {d_\alpha } \right)}},\end{equation*}

where the denominator is the standard deviation of the bootstrap distribution of $R_k \left( {d_\alpha } \right)$ and $R_k^0 \left( {d_\alpha } \right)$ represents the null reproducibility in *B* random permutations across the whole data set. Specifically, ROTS maximizes the reproducibility statistic $Z_k \left( {d_\alpha } \right)$ over a dense lattice of parameters $\alpha$ where $\alpha _1 \ge 0$ and $\alpha _2 \in \left\{ {0,1} \right\}$ and various numbers of top ranked genes between 5 and $G$, where $G$ denotes the total number of genes in the experiment.

The output of ROTS is the optimized *Z*-score, reproducibility and ROTS-statistic for each gene together with false discovery rate (FDR) estimate to assess the significance of differential expression. In RNA-seq studies, small numbers of replicate samples and large biological variation remain the main challenges which call the efficiency of statistical methods under question. With ROTS, the optimized *Z*-score and reproducibility are the main indicators to decide the success of differential expression detection. As a rule of thumb, reproducibility *Z*-scores below 2 indicate that the data or the statistics are not sufficient for reliable detection.

### Other differential expression analysis tools

All the statistical analyses in this manuscript were performed using R version 3.0.2. For comparing ROTS with other available methods, we used edgeR (version 3.2.4), DESeq (version 1.12.1), DESeq2 (version 1.0.19), Cuffdiff 2.0.2, Limma (version 3.16.7), baySeq (version 1.12.0), NOISeq (version 2.12.0) and PoissonSeq (version 1.1.2).

### ccRCC prognosis analysis

Unsupervised clustering was performed by applying the R *hclust* function using the Ward method and Manhattan distances. The patient-specific risk scores were calculated similarly as by Shaughnessy et al. (24). Specifically, the risk scores were defined as the difference between the log_2_-transformed expression levels of the up- and down-regulated genes in the prognostic signature of 152 ROTS detections. Next, the scores were clustered into four groups using K-means clustering method. Finally, the Kaplan–Meier analysis was performed to compare the survival of the ccRCC patients in the four risk categories. The significance of the differences between the categories was tested using the log rank test.

## RESULTS AND DISCUSSION

### ROTS outstanding performance: highest accuracy and lowest false discovery rate

The improved sensitivity and specificity of ROTS over state-of-the-art methods was demonstrated in a controlled spike-in study. Spike-in data sets are benchmarks to investigate the strengths and weaknesses of new computational methods. The spiked data set presented by Rapaport et al. (11), previously used to evaluate different methods for RNA-seq differential expression analysis, was analyzed using ROTS and a number of state-of-the-art methods of the field, including edgeR (5,6), DESeq (7), DESeq2 (25), Limma (26,27), Cuffdiff2 (9), PoissonSeq (28), NOISeq (29) and baySeq (8). This data set includes technical replicates of human whole body (*n* = 5) and human brain samples (*n* = 5) spiked with 92 synthetic oligonucleotides provided by the External RNA Controls Consortium (ERCC) (refer to MATERIALS AND METHODS for details). The pre-determined fold-changes of the synthetic RNAs (4, 1, 0.67 and 0.5) enable measurement of true positive (sensitivity) and true negative detection rates (specificity) of different gene ranking methods.

**Figure 1.**
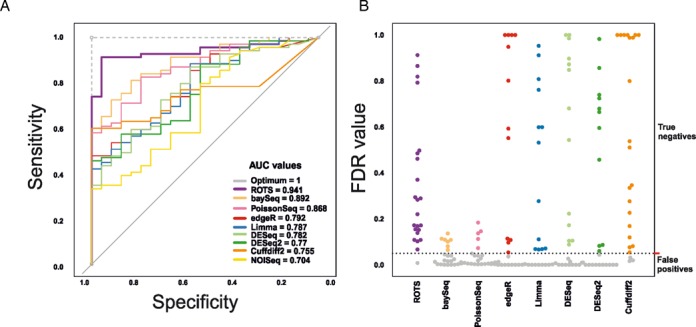
Efficient detection of spiked material. **(A)** Receiver Operating Characteristic (ROC) curves of the different statistical methods in the spike-in data together with the areas under the curves (AUC). **(B)** False discovery rate (FDR) values of the non-differentially expressed spiked controls. The gray dots correspond to false positive detections at FDR < 0.05.

The Receiver Operating Characteristic (ROC) analysis showed how ROTS outperforms the other methods in terms of sensitivity and specificity (Figure 1A; area under the curve AUC = 0.941; DeLong's test *P* < 0.01 compared to all other methods except for baySeq for which *P* = 0.077). The second best method was baySeq (AUC = 0.891), whereas NOISeq showed the lowest AUC value (AUC = 0.704). This comparison was repeated for smaller sample sizes (*N* = 2, 3 and 4) with selected methods (edgeR, DESeq, Limma and ROTS). Again ROTS performed significantly better than the other methods with sample sizes larger than 2 (Wilcoxon rank sum test *P*-value < 0.06; see Supplementary Figure S1). To further investigate the ability of the methods to control type I error rate (i.e. to avoid false positive detections) the approach of the original paper (11) was followed. Specifically, the false discovery rate (FDR) values of the spiked controls (fold-change 1) were examined. They represent non-differentially expressed genes whose FDR values should be high. Strikingly, ROTS showed an outstanding outcome in this comparison (Figure 1B). Among the 23 non-differentially expressed RNA controls, ROTS detected only one false positive at FDR < 0.05, whereas most of the other methods detected at least ten false positives. PoissonSeq presented the worst performance in this comparison with as many as 18 false positive detections. NOISeq was not included in this step as it does not report FDR values for the technical replicates. Our results in the spike-in data set strongly support the significant advantage of ROTS over the current widely used statistical methods.

### ROTS defines prognostic signature of renal cell cancer using two independent data sets

The efficiency of ROTS was then investigated in real patient data involving large biological heterogeneity to take a step toward clinical applications of RNA-seq. The aim was to identify prognostic markers for clear cell renal cell carcinoma (ccRCC), which is an important clinical problem. The ccRCC accounts for the majority of cases of kidney cancer (30). The clinical course of ccRCC is heterogeneous as is the mutational profile. Current prognostic post-nephrectomy markers (e.g. UICC and SSIGN) are based on clinicopathological features such as grade and TNM classification, but ccRCC lacks widely accepted genetic markers for prognosis (31,32). In addition to understanding ccRCC molecular characteristics, accurate biomarkers are required to stratify the disease for selecting patients for adjuvant trials and close surveillance. Recent data from two large published ccRCC studies (18,19), which included both RNA-seq measurements as well as corresponding clinical information, were analyzed. Data from the study by The Cancer Genome Atlas (TCGA) (18) was utilized to detect candidate markers associated with patient outcome (poor or better prognosis) and the findings were verified in a completely independent data by Sato et al. (19) (referred to as validation data in the following).

In the TCGA data, ROTS detected 2208 differentially expressed genes at FDR < 0.05 between 40 patients (∼10%) with the longest survival time (>60 months) and 40 patients with the shortest survival time (<12 months); see Supplementary Table S1 for the characteristics of the groups. The ROTS reproducibility values indicated appropriate reproducibility of the results (*R* = 0.57, *Z* = 5.27). To focus on the most promising candidate markers for ccRCC prognosis, those 152 genes that showed log_2_ fold-change above 1.6 (∼3-fold change) and average expression above the lowest 30% were retained for subsequent analysis, similarly as in the RNA-seq assessment studies (1,3–4) (Supplementary Table S2). For comparison, Limma detected 130 genes with the same criteria, all of which were among the ROTS detections. The genes detected exclusively by ROTS included, for instance, *EPO*, *REN*, *FABP1* and *IGFBP1* that are regulated by the pVHL-HIF pathway, which is commonly overactivated in ccRCC. This supports the potential relevance of the additional ROTS findings.

**Figure 2.**
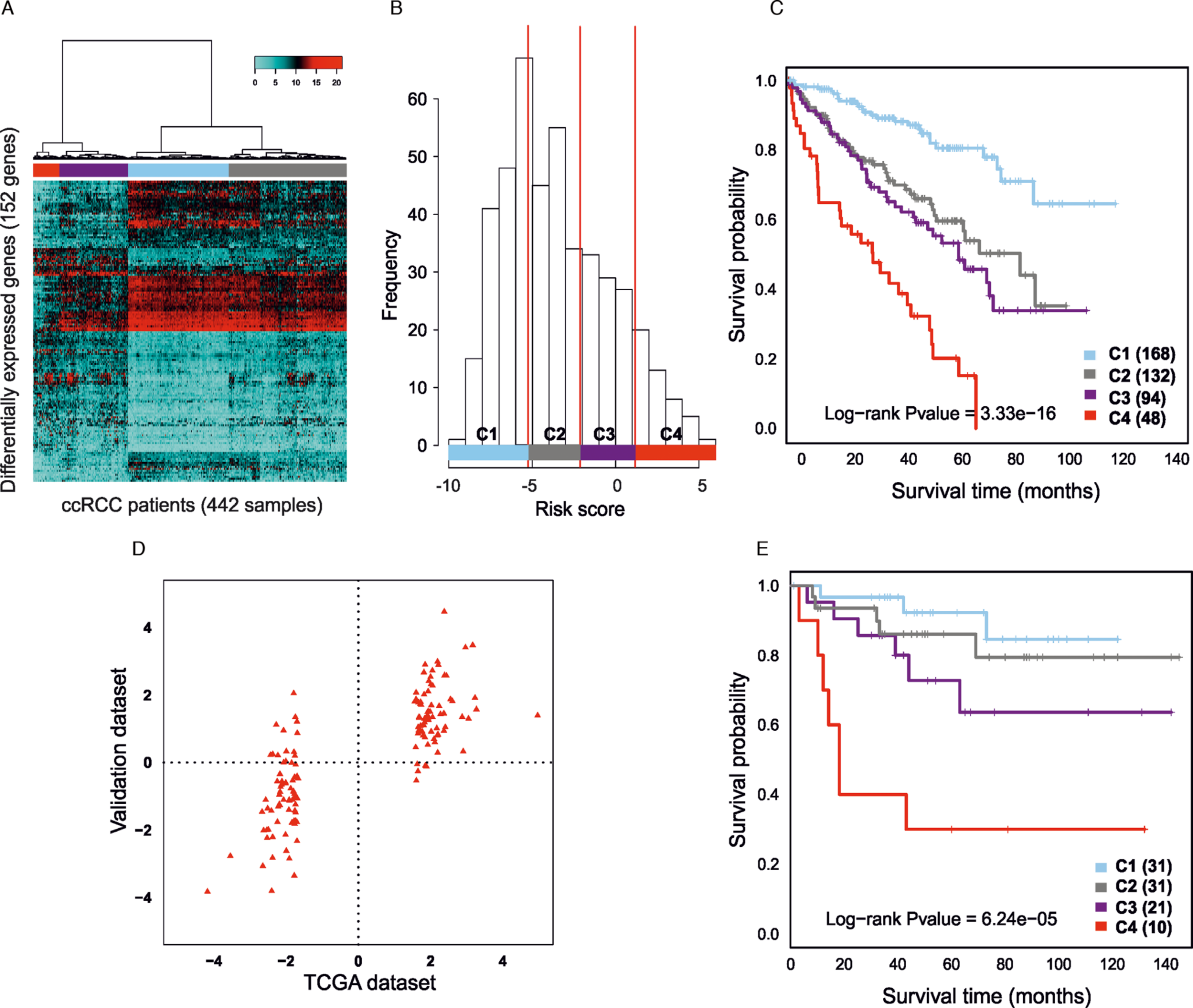
Novel prognostic markers for ccRCC. **(A)** Unsupervised clustering of the patients (columns) across the genes detected as differentially expressed by ROTS (rows). **(B)** Distribution of the risk scores across the patients in the TCGA data. The red vertical lines show the cutoffs used to define the four risk categories (−5.10, −2.17, 0.94). The risk categories significantly overlapped with the similarly colored clusters in panel A (69, 92, 78 and 92 percent of overlap with C1, C2, C3 and C4 survival groups respectively; Fisher's exact test *P*-value < 0.01). **(C)** Kaplan–Meier curves comparing the survival of the ccRCC patients in the four risk categories in the TCGA data. The numbers in parentheses indicate the numbers of patients in the different risk categories. The colors correspond to the colors in panels A and B. **(D)** Correlation of the signal log-ratios of the differentially expressed genes (red triangles) between the best and poorest survival patients in the TCGA (*x*-axis) and validation data (*y*-axis). Patients with comparable survival times (<12 months, >60 months) were considered in the analysis. A highly significant Pearson correlation of 0.796 was observed (*P* < 10^–15^). **(E)** Kaplan–Meier curves comparing the survival of the ccRCC patients in the four risk categories in the validation data. The highly significant association between the risk score categories and survival verify the risk score model developed using the TCGA data.

To assess the utility of the 152 ROTS detections as prognostic markers, a risk score was defined for each of the 442 patients in the complete TCGA data in terms of signal log-ratio of the up-regulated versus the down-regulated genes (24,33) (refer to MATERIALS AND METHODS for details). Investigation of the expression levels of the detected genes revealed four clusters of ccRCC patients (Figure 2A). Accordingly, four risk score categories were defined (Figure 2B). This revealed a highly significant association between the risk scores and the survival of the patients (Figure 2C; log rank test *P* < 10^−15^). While the 5-year survival in the best group (blue) was ∼80%, it decreased below 20% in the worst survival group (red); in the two intermediate groups the 5-year survival was 50–60%. This supports the prognostic value of the detected genes beyond the 80 samples used for detecting the markers.

To avoid over-fitting to a single study, further validation of the markers and risk score model was carried out in a completely independent data set of 100 ccRCC patients (19). The signal log-ratios of the detected genes between the best and poorest survival patients were highly correlated across the TCGA and validation data when patients with comparable survival times (<12 months, >60 months) were considered (Figure 2D; Pearson correlation 0.796, *P* < 10^−15^). Over 90% of the detected 152 genes showed a concordant change to the same direction in both data sets (Supplementary Table S2, Supplementary Figures S2 and S3). Importantly, a highly significant association between the risk scores and survival verified the risk score model developed using the TCGA data (Figure 2E; log rank test *P* < 10^−4^). These results confirm the capability of ROTS to robustly identify reproducible candidate markers well as the potential of the risk score model to identify especially poor prognosis ccRCC patients.

### Biological and technical evaluation of detected prognostic biomarkers and utilized method

**Figure 3.**
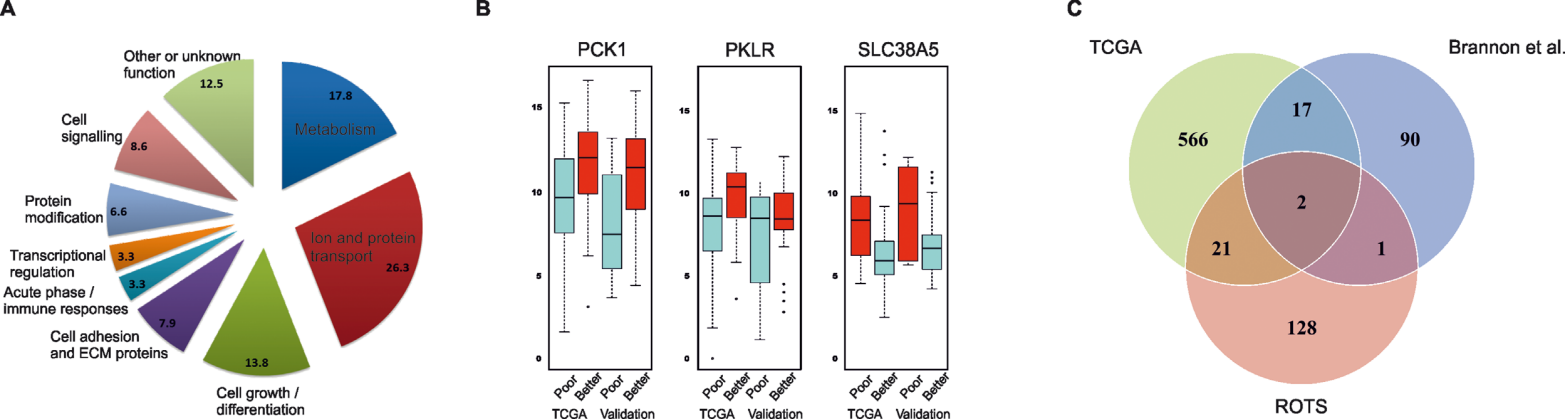
Biological insights from ccRCC prognostic markers. **(A)** Functional groups of the differentially expressed genes detected by ROTS. **(B)** Examples of the detected differentially expressed genes. The boxes show the median and the interquartile range (IQR) of the expression levels of the poor and better prognosis patients in the TCGA and validation data, the whiskers indicate their range and the points correspond to extreme observations with values greater than 1.5 times the IQR. The boxplots for all the differentially expressed genes are shown in Supplementary Figures S2 and S3. **(C)** Venn diagram summarizing the overlap between the prognostic genes reported by the previous studies (18,36) and ROTS (see Supplementary Table S2 for detailed information).

Analysis of the detected genes using the Ingenuity Pathway Analysis (IPA) tool suggests three major functional groups: molecular transport, small molecule biochemistry, and amino acid and lipid metabolism (Supplementary Table S3; *P* < 0.05). Categorization of the genes into five main biological function groups and four additional biochemical function groups implies major involvement of metabolism (∼18% of the genes), particularly glucose metabolism, as found also in the original TCGA study (Figure 3A, Supplementary Table S4). Several previously reported markers were confirmed such as the key glucose metabolism regulators *ALDOB*, *G6PC* and *PKLR* (Figure 3B). Additionally, several new metabolism regulating markers were detected that have been missed by previous large-scale studies. These include, for instance, the glucose transporter/sensor *SLC2A2*, and the central gluconeogenesis regulator *PCK1* (Figure 3B).

The largest proportion (∼26%) of the prognostic genes were from the cellular transporter and solute carrier groups (Figure 3A). The solute carrier family genes were highly enriched; 26 (∼9%) out of ∼300 family members were among the prognostic genes (Fisher's exact test *P* < 0.05). Some of them, such as members of solute carrier family 16, were detected by the TCGA study. Additionally, ROTS detected 19 previously undetected solute carrier genes, including for example *SLC38A5*, which transports glutamate, an essential nitrogen donor for cancer cells to build amino acids and to maintain mTOR activity (Figure 3B).

The better prognosis genes included most of the detected glucose metabolism and organic anion/cation transporters. Increased expression of six glycolytic genes were categorized into good prognosis group (ALDH1L1, ALDOB, G6PC, PCK1, PKLR, SLC2A2). The glycolytic genes are direct transcriptional targets of HIF-1α, which is known to function as a tumor suppressor in kidney cancer in contrast to several other cancer types (34,35). The finding is in line with previously reported metabolic shift in RCC and increased feed of the TCA cycle in good prognosis group (18). (Supplementary Figure S4). Network analysis of the ROTS detections in the manually curated Ingenuity Knowledge Base revealed several interactions between the better prognosis genes linked to cellular metabolism (Supplementary Figure S5). These observations are in line with the earlier view (18). The poor prognosis genes were enriched in a variety of cell growth signaling molecules (e.g. phosphatases), extracellular matrix and remodeling proteins (collagens, metalloproteins) and acute phase/immune response genes (CRP, SAA family) (Supplementary Figure S4).

Comparison of the ROTS detections to two previous studies (18,36) of prognostic markers for ccRCC revealed a statistically significant but moderate overlap (Figure 3C and Supplementary Table S2; Fisher's exact test *P* < 0.05). For instance, the overlapping genes included the *ACE2* and *NPR3* that regulate blood pressure by the renin-angiotensin system. Additionally, new genes within this pathway were detected, including *REN*, the primary regulator of the pathway secreted by kidney cells. To further validate the method we studied the association of several known genes mutated in RCC with our prognostic groups (Supplementary Figure S6). In line with previous reports ROTS predicted PBRM1 mutations to correlate well with good prognosis (*P* < 0.01) (18,37). Also, BAP1 mutations revealed statistically significant correlation with poorer prognosis (*P <* 0.01). Several other mutated genes were also tested for prognostic correlation (e.g. TP53, CDKN2A and PIK3CA) but their mutation rate was too low to reveal any significant correlation. Moreover, our algorithm predicted a strong trend toward better prognosis in the VHL mutated group (*P* < 0.05). Although studies attempting to correlate VHL mutation with patient prognosis have been somewhat conflicting, correlation of wild-type VHL with poor prognosis has been reported earlier (38,39).

Taken together, our results demonstrate the utility of the RNA-seq technology to detect reproducible markers when an appropriate test statistic is applied. Systematic tools are needed for unbiased and effective analysis of RNA-seq data to fulfill the high promise posed by the technology. Instead of developing new variants of different statistical tests, users require practical tools to choose an optimal method for their own data. The reproducibility optimization procedure enables this. Additionally, it provides information about the quality of the detections; low reproducibility values indicate that the data or the test statistics are not sufficient for reliable detection.

The high validation percentage of the detected ccRCC prognostic markers in independent data supports the general potential of ROTS in clinical RNA-seq studies. Although the main focus was on reliable marker detection, the developed ccRCC risk score model illustrates the use of the detections for disease signatures. The identified novel candidate genes serve as good starting points for further validation studies to confirm their utility in the clinic as support tools to predict ccRCC prognosis or revealing novel potential targets for ccRCC treatment. Our results suggest that ROTS enables stratification of patients in prognostic groups that can help to select patients for future RCC adjuvant trials and closer post-nephrectomy follow-up to timely reveal metastatic disease. The identified markers imply high potential of genes regulating cell growth and metabolism but also ion transport apart from glucose transport. It is also noteworthy that four genes regulating blood pressure through the renin-angiotensin system were identified as good prognosis markers, as blood pressure is commonly elevated in RCC patients and functions as a surrogate marker for tyrosine kinase inhibitor treatment efficacy.

## AVAILABILITY

ROTS package is implemented with R and the source code together with Windows and Mac OS binaries are freely available at http://www.btk.fi/research/research-groups/elo/software/rots/. In order to make the study fully reproducible, we provide all the codes to run the analyses in the Supplementary Code, which provides detailed information about the methods and parameters used in this study.

## Supplementary Material

SUPPLEMENTARY DATA
