# Supplementary material for: ROTS: reproducible RNA-seq biomarker detector—prognostic markers for clear cell renal cell cancer
Source: Nucleic Acids Res. 2015 Aug 11;44(1):e1. doi: 10.1093/nar/gkv806 (PMC4705679; doi:10.1093/nar/gkv806)
Supplement: SUPPLEMENTARY DATA [file supp_44_1_e1__index.html]

ROTS: reproducible RNA-seq biomarker detector—prognostic markers for clear cell renal cell cancer — ROTS: reproducible RNA-seq biomarker detector—prognostic markers for clear cell renal cell cancer — SUPPLEMENTARY DATA 

# ROTS: reproducible RNA-seq biomarker detector—prognostic markers for clear cell renal cell cancer

## SUPPLEMENTARY DATA

- SUPPLEMENTARY DATA
