# Supplementary material for: ROTS: reproducible RNA-seq biomarker detector—prognostic markers for clear cell renal cell cancer
Source: Nucleic Acids Res. 2015 Aug 11;44(1):e1. doi: 10.1093/nar/gkv806 (PMC4705679; doi:10.1093/nar/gkv806)
Supplement: SUPPLEMENTARY DATA [file supp_gkv806_Supplementary_06_08_2015.zip › Elo_Supplementary.pdf]

Supplementary Information

ROTS: reproducible RNA-seq biomarker detector – prognostic markers for clear cell renal cell cancer

Fatemeh Seyednasrollah[1,4], Krista Rantanen[1,2], Panu Jaakkola[1,2,3], Laura L. Elo[1,4]

[1] Turku Centre for Biotechnology, University of Turku and Åbo Akademi University, Turku, FI-20520, Finland
[2] Department of Medical Biochemistry, University of Turku, Turku, FI-20014, Finland
[3] Department of Oncology and Radiotherapy, Turku University Hospital, FIN-20520 Turku, Finland
[4] Department of Mathematics and Statistics, University of Turku, Turku, FI-20014, Finland

Abstract

Recent comprehensive assessments of RNA-seq technology support its utility in quantifying gene expression in various samples. The next step of rigorously quantifying differences between sample groups, however, still lacks well defined best practices. Although a number of advanced statistical methods have been developed, several studies demonstrate that their performance depends strongly on the data under analysis, which compromises practical utility in real biomedical studies. As a solution, we propose to use a data-adaptive procedure that selects an optimal statistic capable of maximizing reproducibility of detections. After demonstrating its improved sensitivity and specificity in a controlled spike-in study, the utility of the procedure is confirmed in a real biomedical study by identifying prognostic markers for clear cell renal cell carcinoma (ccRCC). In addition to identifying several genes previously associated with ccRCC prognosis, several potential new biomarkers among genes regulating cell growth, metabolism and solute transport were detected.

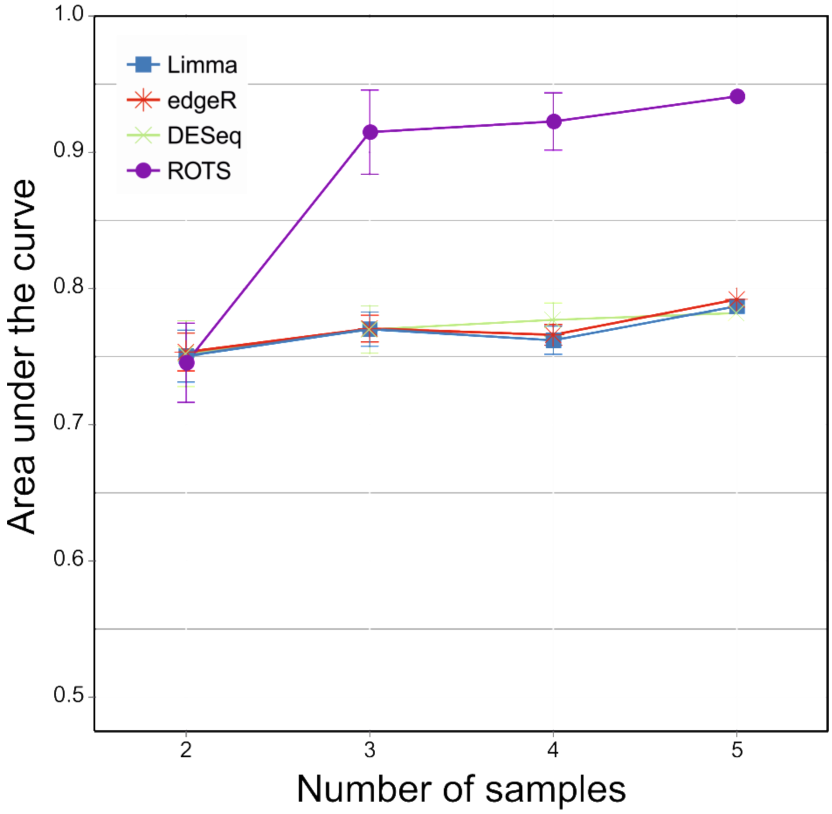

Supplementary Figure S1. Area under the curve (AUC) of ROC for selected statistical methods with different sample sizes N = {2,3,4,5} in the spike-in data.

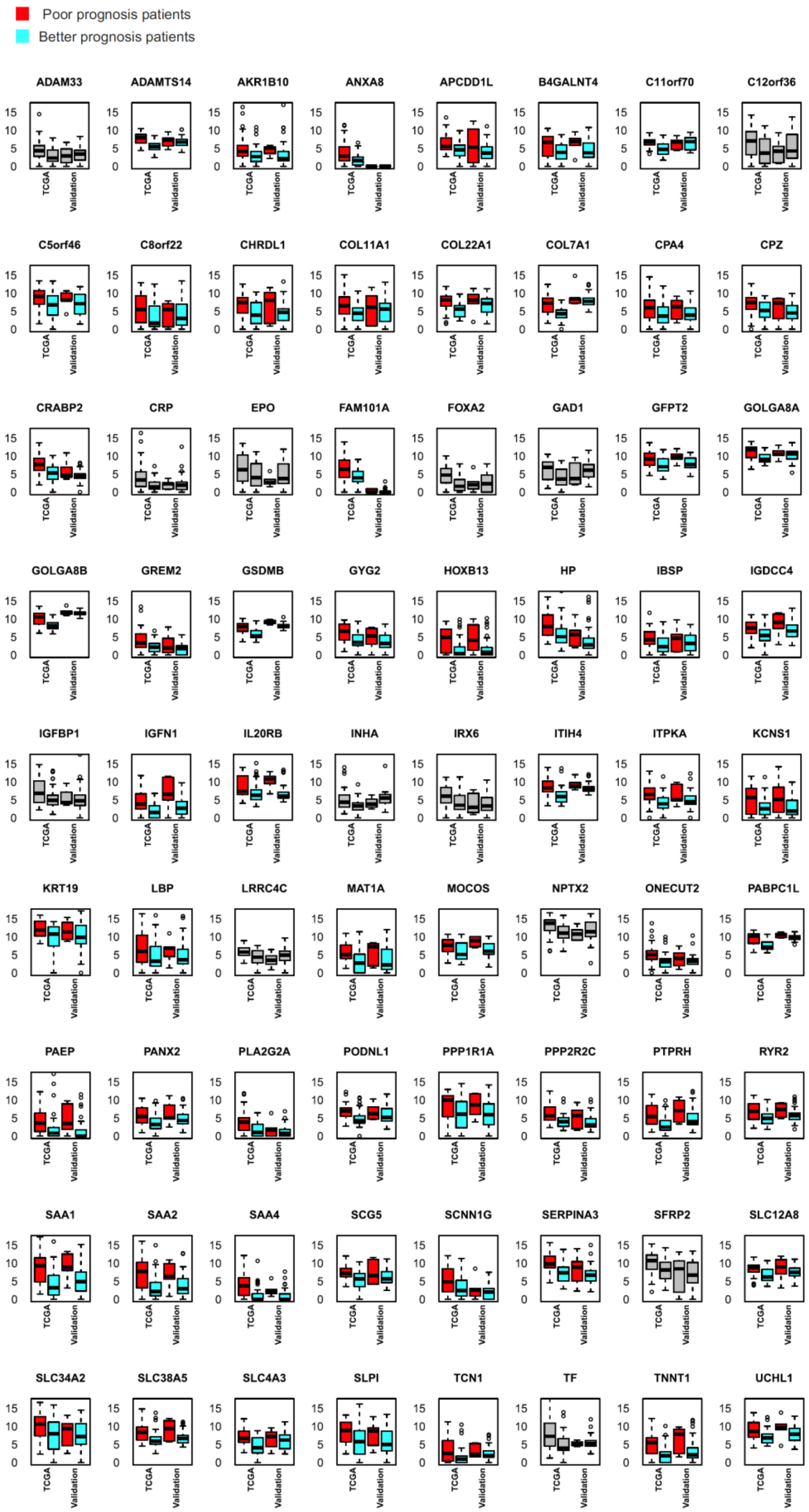

Supplementary Figure S2. Boxplots of the genes up-regulated in the poor prognosis patients in the TCGA data. The boxes show the median and the interquartile range (IQR) of the expression levels of the poor and better prognosis patients in the TCGA and validation data, the whiskers indicate their range, and the points correspond to extreme observations with values greater than 1.5 times the IQR. Gray color indicates genes with discordant signal log-ratios between the TCGA and validation data.

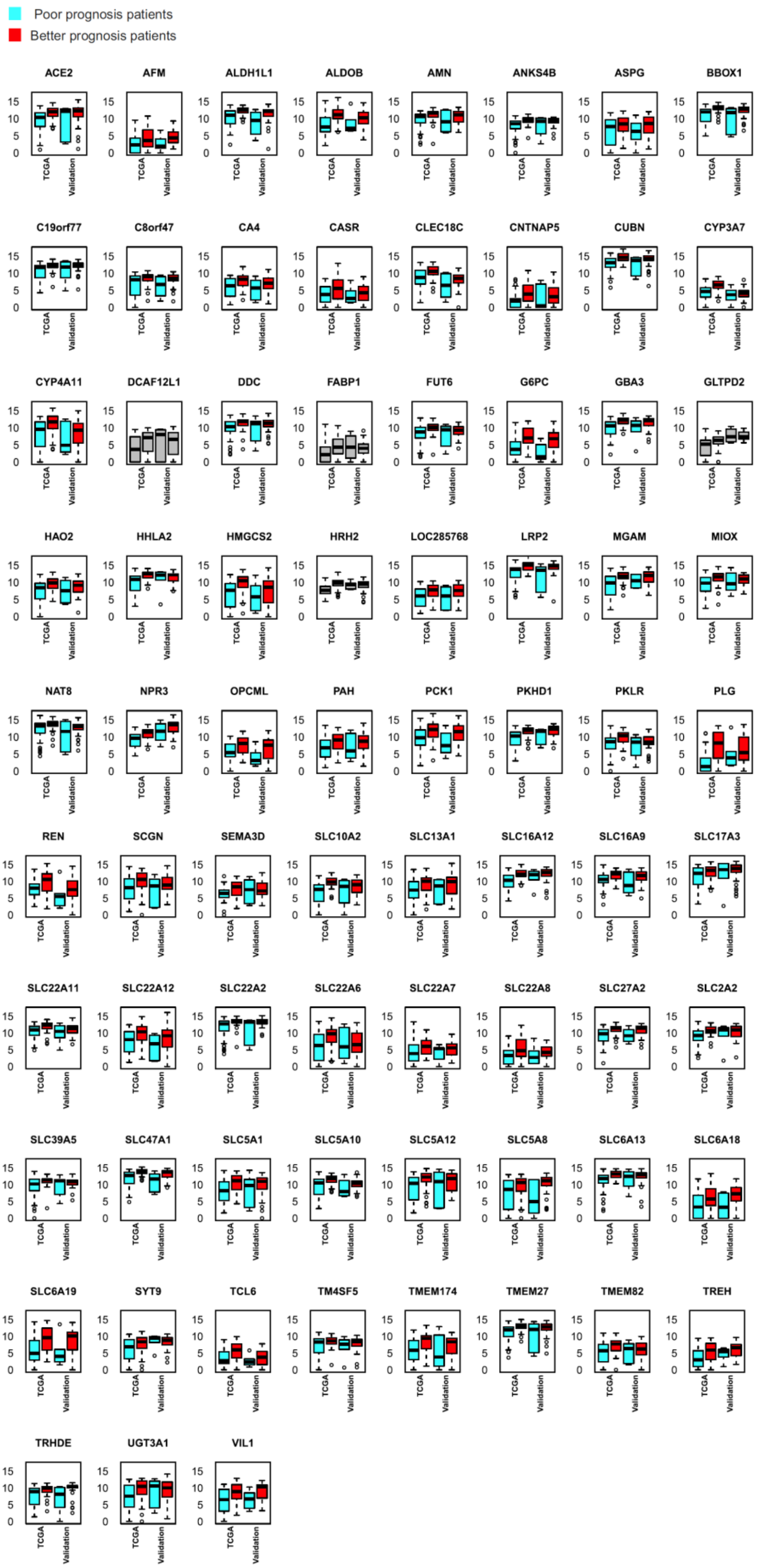

Supplementary Figure S3. Boxplots of the genes up-regulated in the better prognosis patients in the TCGA data. The boxes show the median and the interquartile range (IQR) of the expression levels of the poor and better prognosis patients in the TCGA and validation data, the whiskers indicate their range, and the points correspond to extreme observations with values greater than 1.5 times the IQR. Gray color indicates genes with discordant signal log-ratios between the TCGA and validation data.

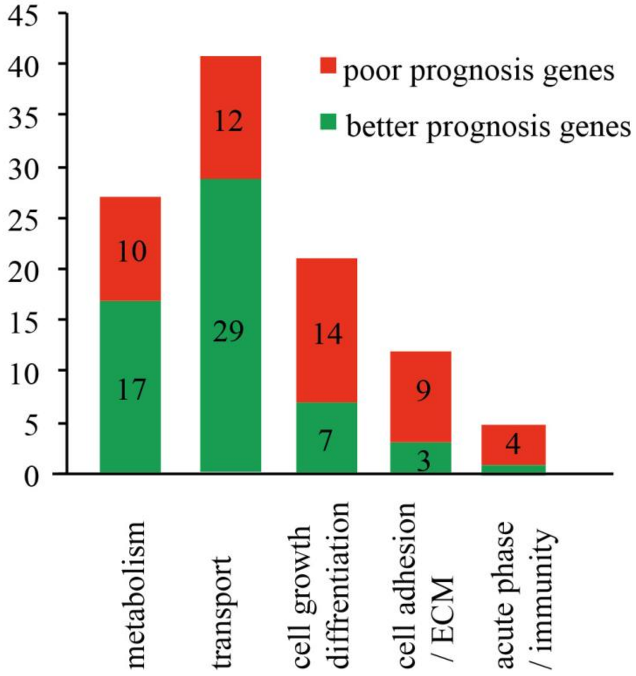

Supplementary Figure S4. Number of genes showing higher expression in poor (red) or better (green) prognosis patients in five main biological function groups.

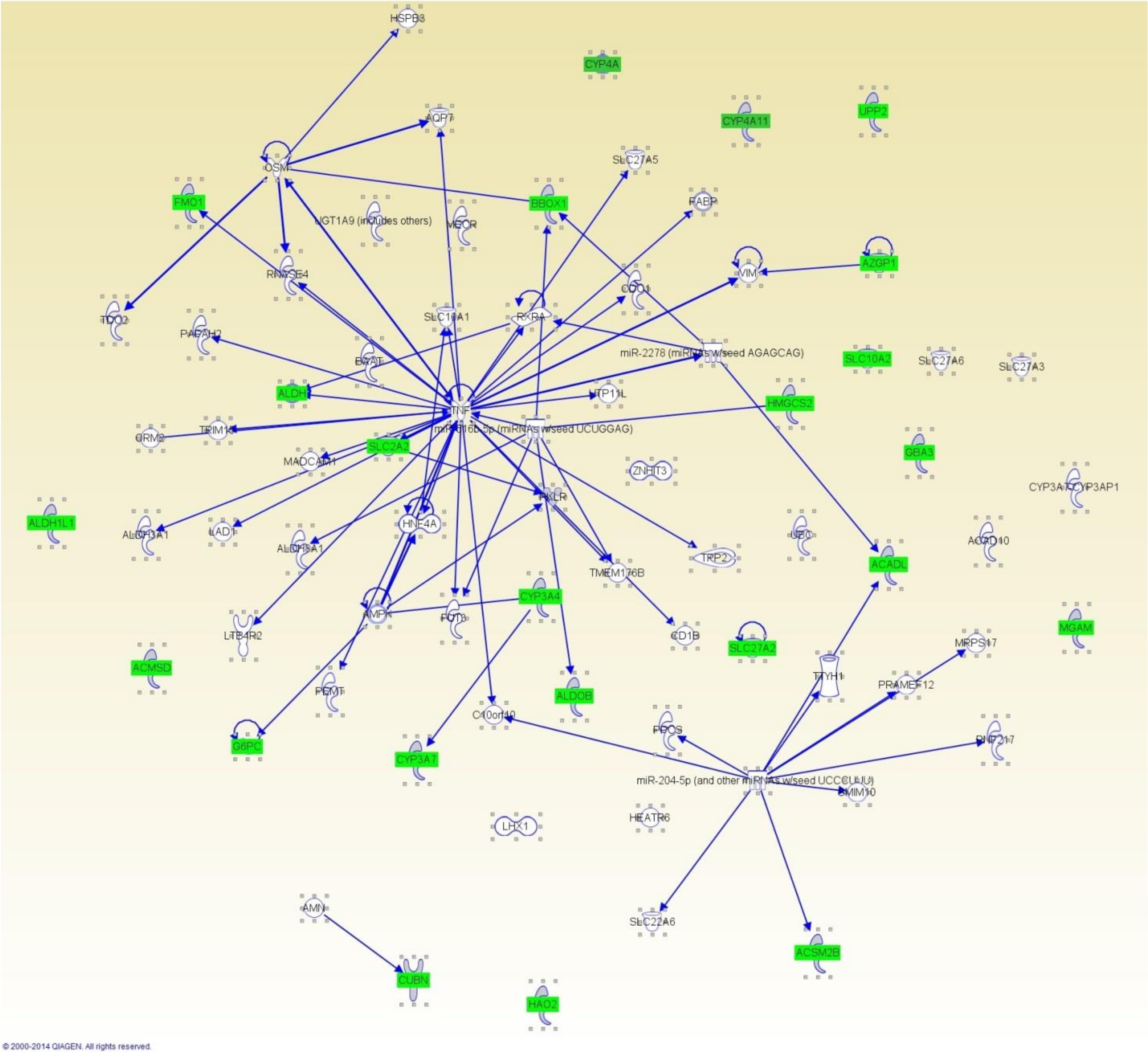

© 2000-2014 QIAGEN. All rights reserved.

Supplementary Figure S5. Interaction network connecting better prognosis genes linked to cellular metabolism. The network analysis was done using the Ingenuity Pathway Analysis (IPA) tool. The solid lines indicate direct interactions. Genes detected by ROTS are shown in green.

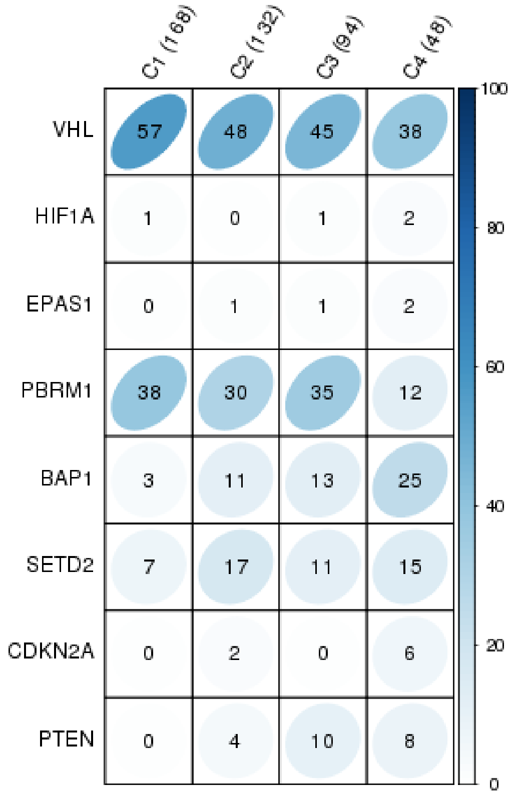

Supplementary Figure S6. Mutation status of TCGA patients for a set of selected genes. The numbers show the percentage of patients with mutation for each specified survival group.
